# High C3 photosynthetic capacity and high intrinsic water use efficiency underlies the high productivity of the bioenergy grass *Arundo donax*

**DOI:** 10.1038/srep20694

**Published:** 2016-02-10

**Authors:** Richard J. Webster, Steven M. Driever, Johannes Kromdijk, Justin McGrath, Andrew D. B. Leakey, Katharina Siebke, Tanvir Demetriades-Shah, Steve Bonnage, Tony Peloe, Tracy Lawson, Stephen P. Long

**Affiliations:** 1 Institute of Biological Environmental and Rural Sciences, Aberystwyth University, Aberystwyth, U.K; 2 Centre for Crop Systems Analysis, Wageningen University, Wageningen, the Netherlands; 3 Department of Plant Biology, University of Illinois at Urbana-Champaign, Urbana, Illinois, USA; 4 Carl R. Woese Institute for Genomic Biology, University of Illinois at Urbana-Champaign, Urbana, Illinois, USA; 5 Heinz Walz GmbH, Effeltrich, Germany; 6 LI-COR Biosciences UK Ltd., Cambridge, UK; 7 ADC BioScientific Ltd., Hoddesdon, UK; 8 Delta-T Devices Ltd., Cambridge, UK; 9 School of Biological Sciences, University of Essex, Wivenhoe Park, Colchester, U.K

## Abstract

*Arundo donax* has attracted interest as a potential bioenergy crop due to a high apparent productivity. It uses C3 photosynthesis yet appears competitive with C4 grass biomass feedstock’s and grows in warm conditions where C4 species might be expected to be that productive. Despite this there has been no systematic study of leaf photosynthetic properties. This study determines photosynthetic and photorespiratory parameters for leaves in a natural stand of *A. donax* growing in southern Portugal. We hypothesise that *A. donax* has a high photosynthetic potential in high and low light, stomatal limitation to be small and intrinsic water use efficiency unusually low. High photosynthetic rates in *A. donax* resulted from a high capacity for both maximum Rubisco (*V*_c,max_ 117 μmol CO_2_ m^−2^ s^−1^) and ribulose-1:5-bisphosphate limited carboxylation rate (*J*_max_ 213 μmol CO_2_ m^−2^ s^−1^) under light-saturated conditions. Maximum quantum yield for light-limited CO_2_ assimilation was also high relative to other C3 species. Photorespiratory losses were similar to other C3 species under the conditions of measurement (25%), while stomatal limitation was high (0.25) resulting in a high intrinsic water use efficiency. Overall the photosynthetic capacity of *A. donax* is high compared to other C3 species, and comparable to C4 bioenergy grasses.

Giant reed (*Arundo donax* L.) has attracted interest as a potential bioenergy crop, due to a high apparent productivity and suitability as an accepted feedstock for cellulosic fuel production^1^. It is found throughout the Mediterranean climate zones of the world and has been cultivated in Asia, southern Europe and North Africa for over a thousand years. It is a rhizomatous perennial herbaceous grass that produces stems of ca. 2–3 cm diameter that may grow to heights of 3–6 m. It is found in moist grasslands and wetlands although it is able to thrive across a wide range of soil types, while also being tolerant of mild drought and salinity, and is tolerant of water-logged soils^2^,^3^,^4^. In climates with warm winters it is evergreen but dies back in climates with freezing winter temperatures. Although there are related species within the genus, molecular analysis suggests that *A. donax* is monophyletic in origin and its spread has not involved hybridisation with the related species. These analyses also suggest that it originated in west Asia and then spread to the Mediterranean region^5^. The high productivity of *A. donax* is achieved in warm growing conditions despite the fact that it uses C3 photosynthesis, which might be expected to be a competitive disadvantage compared to C4 species under such growing conditions.

Side-by-side trials in central Italy over 11 years showed an average yield of above-ground dry biomass of 28.7 t ha^−1^ yr^−1^ for the highly productive C4 perennial grass *Miscanthus x giganteus* Deu et Greef, but 37.7 t ha^−1^ yr^−1^ for *A. donax*^6^. Although this appears a relatively low yield for *M. x giganteus* under irrigated conditions in the Mediterranean^7^, the reported yield for *A. donax* is high by any measure. *A. donax* in central and southern Italy showed average dry matter yield over 3 years of up to 74 t ha^−1^ yr^−1^, which exceeds the highest yields reported for *M. x giganteus*^8^. Similarly, in a multi-year study in Alabama *A. donax* achieved an average yield of 35.5 t ha^−1^ yr^−1^ compared to 23.5 t ha^−1^ yr^−1^ for switchgrass (*Panicum virgatum* L.)^9^. In a comparison with C4 sweet sorghum (*Sorghum bicolor* L.) in northern Italy, light penetration into an *A. donax* canopy studied over a three year period was found to be 60% higher and the efficiency of conversion of intercepted radiation into biomass was *ca*. 60% higher. This might be explained by its more effective distribution of light through the canopy, if it is coupled with a similar efficiency of leaf photosynthesis^10^,^11^. However, yields may be considerably lower on marginal land. On a sandy loam with limited nutrient availability *A. donax* required three years to accumulate 20 t ha^−1^, and build a rhizome mass of 16 t ha^−1^ 4, by contrast to *M. x giganteus* which has proved significantly more productive on marginal sites^12^. While *M. x giganteus* was found particularly vulnerable to damage by water-logging of soils during autumn and winter, *A. donax* appeared to thrive in these situations. This tolerance of water-logging also allows *A. donax* to spread along watercourses from rhizome fragments where *M. x giganteus* cannot^13^. Its ability to survive anaerobic soil may explain its ability to tolerate high levels of reduced ions of heavy metals^14^. *A. donax* is clearly a resilient, but also very productive species. However, there is little fundamental knowledge available on how it is able to realise this productivity. Understanding this demands knowledge of leaf photosynthesis as well as canopy microclimate.

Leaf photosynthetic rates of CO_2_ uptake (*A*) measured in *A. donax* growing on an estuary in S. Africa were between 20 and 37 μmol m^−2^ s^−1^ 15. By combining modulated chlorophyll fluorescence and gas exchange, photorespiration was estimated to decrease *A* by 30%. Although water potential declined to −2.1 MPa around noon on sunny days, stomatal conductance (*g*_s_) and *A* changed little. Such low leaf water potential would normally be expected to at least cause partial stomatal closure in most species^15^. The rates of stomatal conductance reported are at the upper end of those found in other C3 species but not much greater than those of the productive C4 bioenergy grasses *M. x giganteus* and *P. virgatum*, as well as productive modern cultivars of maize (*Zea mays* L.)^16^,^17^.

In summary, *A. donax* achieves and exceeds the productivities normally associated with C4 perennial grasses in warm climates. For a plant which forms a high leaf area index (LAI) monocultures in nature and as a crop, it would be expected to have a high photosynthetic capacity at both high and low light intensity under warm conditions. The high photosynthetic capacity is needed in the context of a dense canopy where shaded leaves need to achieve high efficiency, as well as those in full sun, and where all leaves need to be efficient under the low light conditions of dawn, dusk and cloudy days. Yet it is a C3 species, growing in warm conditions where only a C4 species might be expected to be photosynthetically efficient^18^. The limited published data suggests that *A. donax* may indeed have high light-saturated leaf photosynthetic rates, which may also be supported by high leaf conductances. High leaf conductances will allow a higher CO_2_ concentration at ribulose-1:5-bisphosphate carboxylase/oxygenase (Rubisco), so increasing the rate of carboxylation and decreasing photorespiration, but at the expense of water loss and water use efficiency^19^.

To better define the photosynthetic capacity of *A. donax* and understand how this may explain its high productivity this study aimed to define key *in vivo* properties. These were: *J*_max_, the maximum whole chain electron transport rate supporting ribulose-1:5-bisphosphate (RuBP) regeneration, *V*_c,max_ the maximum rate of carboxylation that can be supported by Rubisco, the light-saturated rate of CO_2_ assimilation (*A*_sat_) and the maximum quantum yield of CO_2_ assimilation (Φ_CO2,max_) defined by the initial slope of the response of *A* to absorbed photon flux (*αI*). Concurrent measurement of water vapour flux and modulated chlorophyll fluorescence were used to determine *g*_s_, stomatal limitation to CO_2_ uptake (*l*), intercellular CO_2_ concentration (*c*_i_), and the whole chain electron transport rate (*J*). These parameters were determined for leaves in a natural stand of *A. donax* growing in a dry valley in S. Portugal. These are used to test the hypotheses that *A. donax* has a high photosynthetic potential in both high and low light, that stomatal limitation may be unusually small and thus intrinsic leaf water use efficiency poor. The *in vivo* measures: *J*_max_, *V*_c,max_, *A*_sat_ and Φ_CO2,max_, are key to parameterization of the steady-state biochemical model of C3 photosynthesis^20^ which underlies most models of crop and ecosystem productivity^21^. This study therefore also serves as a key resource for parameterizing production models of this emerging crop.

## Materials and Methods

### Field location

The study was undertaken on a naturally occurring *A. donax* stand located at Quinta de São Pedro - Centro de estudos (Armadas, 2815–786 Sobreda, Portugal 38°38′40.6″N 9°11′34.5″W, altitude 85 m). A mixed 5 acre site with a disturbed dry valley community of annuals, patches of native macchia and scrub, pine woodland, introduced Acacia woodland and a few planted ornamentals on a Cambisol soils^22^.

### Meteorological assessment

Relative humidity, air temperature and irradiance were measured over the sampling period using a Weather Station (WS-GP2 with GP2 Data Logger, Delta-T Devices Ltd, Cambridge, UK) (Fig. S1). Relative Humidity and air temperature were used to calculate vapour pressure deficit (VPD), as a virtual channel within the GP2. Soil temperature and soil water content were measured within the stand over the sampling period using soil moisture sensors (SM300 with GP2 Data Logger, Delta-T Devices Ltd, Cambridge, UK).

### Stomatal Conductance and *In situ* Chlorophyll Fluorescence

Measurements of stomatal conductance were made with a diffusion porometer (AP4, Delta-T Devices Ltd, Cambridge, UK). All measurements of conductance and photosynthesis were made on the most recently expanded leaf, as judged by ligule emergences. Readings were taken on several leaves, locating the porometer at three points along each leaf: 1) the base just above the ligule, 2) the midpoint between ligule to tip; and 3) the area just below the leaf tip. Both the adaxial and abaxial surfaces were measured at these points at midday and mid-afternoon following the procedures of Monteith^23^, and Potter^24^. Modulated chlorophyll fluorescence was monitored to estimate the operating efficiency of PSII (*F*_q_′/*F*_m_′)^25^,^26^ on fully emerged leaves at midday and late afternoon using a fluorometer (MONITORING-PAM, Heinz Walz GmbH, Effeltrich, Germany) (Fig. S1).

### Photosynthetic intercellular-CO_2_ response curves

Leaves were sampled pre-dawn from the *A. donax* stand. Leaves were excised just above the ligule, and immediately re-cut under water and then kept in cool low-light conditions (20 °C and 20 μmol m^−2^ s^−2^) until use. The response of net leaf CO_2_ uptake rate (*A*) to external CO_2_ concentration (*c*_a_) was assessed on five different leaves. Leaves were placed in the leaf cuvette of a portable open gas exchange system (LCPro-SD with 6.25 cm^2^ cuvette; ADC BioScientific Ltd. Herts, England; or LI6400XT with 6 cm^2^ cuvette; LI-COR Biosciences, Lincoln, Nebraska, USA). Air temperature was controlled at 25 ^o^C, leaf temperature 27 ^o^C and VPD at 0.96 kPa. To fully induce photosynthesis before measurements commenced, leaves were first allowed to equilibrate at a photon flux (*I*) of 500 μmol m^−2^ s^−1^ and reference *c*_a_ of 400 μmol mol^−1^ until *A* had reached a stable value, the *I* was then increase to 1500 μmol m^−2^ s^−1^. When *A* had reached a stable value at *I* = 1500 μmol m^−2^ s^−1^ *c*_a_ was then changed to the following levels in sequence, 300, 250, 200, 150, 100, 50, 400, 600, 900, 1200 and 1500 μmol mol^−1^. The leaf remained at each level until a stable *A* could be determined. Potential Rubisco carboxylation (*V*_c,max_) and electron transport through photosystem II (*J*_max_) were determined from the responses of *A*_sat_ to *c*_i_. For each leaf values of *A* located above the transition between Rubisco-limited and RuBP- or electron transport-limited photosynthesis in the *A*/*c*_i_ response were used to solve for *J*_max_ using the equations representing RuBP regeneration-limited *A*^20^. Values were temperature adjusted to 25 ^o^C using the temperature response equations of Bernacchi *et al.* (2001)^27^ for the Rubisco-limited, and Bernacchi *et al.* (2003)^28^ for the RuBP regeneration limited portions of the *A* vs *c*_i_ curves. Stomatal limitation (*l*) at the current ambient *c*_a_ of 400 μmol mol^−1^ was derived using the method outlined in Long and Bernacchi (2003)^29^.

### Photosynthetic light response curves

Sampling and measurements of *A* vs. *I* were performed in parallel with *A* vs. *c*_i_ curves. Leaves were placed in the leaf cuvette of an integrated open gas exchange system and modulated chlorophyll fluorometer (GFS-3000FL, LED-Array/PAM-Fluorometer 3055-FL and 3080-O2 Oxygen sensor. Heinz Walz GmbH, Effeltrich, Germany). Leaves were adapted to an incident photon flux of 1500 μmol m^−2^ s^−1^ prior to measurements with the [CO_2_] at 400 μmol mol^−1^ and leaf temperature controlled at 25 ^o^C. The VPD of the air entering the gas exchange system was maintained between 0.8 and 1.1 kPa. Photosynthetic light response curves were then obtained at ambient (21%) and low (2%) oxygen concentrations. Photon flux was varied in a step-wise manner, either starting from the photon flux at which photosynthesis had been induced, after a steady-state *A* was obtained or alternating between different photon fluxes, in each case waiting for a steady-state to be obtained. Photon flux levels were 2000, 1500, 1000, 700, 500, 350, 250, 150, 75, 45 μmol m^−2^ s^−1^. At each light level, once a new steady state was reached gas exchange rates were recorded. Simultaneously, *F*_q_′/*F*_m_′ was determined via modulated chlorophyll fluorescence and application of a saturating flash as previously described^25^,^26^,^30^.

The light response of CO_2_ assimilation rate is described by a four-parameter non-rectangular hyperbola, according to Marshall and Biscoe^31^:


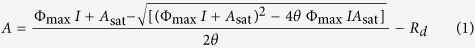


where *A* is the CO_2_ assimilation rate (μmol m^−2^ s^−1^), Φ_max_ is the apparent maximum quantum yield (mol CO_2_ (mol photons)^−1^), *I* is the incident photon flux (μmol m^−2^ s^−1^), *A*_sat_ is the CO_2_ assimilation rate at saturating photon flux (μmol m^−2^ s^−1^), *θ* is the curve convexity (dimensionless) and *R*_d_ is mitochondrial respiration in the light (μmol m^−2^ s^−1^). The light compensation point (LCP) was determined as the incident photon flux (*I*) where *A* = 0 as predicted from the fitted curve.

The quantum yield of CO_2_ assimilation, Φ_*CO2*_, was determined according to Genty^30^:


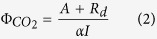


where *A* is corrected for respiratory loss (*R*_d_) and divided by the absorbed photon flux (*αI*), with *α* as the leaf absorbance. The method used to determine absorbance under the chamber lighting conditions is described later.

The parameters Φ_*CO2*_ and *F*_q_′/*F*_m_′ have been shown to be linearly related under conditions where photorespiration is suppressed^30^,^32^,^33^. Under ambient (21%) and low (2%) oxygen atmosphere, the relationship can be used to assess the possible operation of electron acceptors other than CO_2_, e.g. photorespiration or active oxygen production, which are manifest as an increased *F*_q_′/*F*_m_′ relative to its corresponding Φ_*CO2*_^34^.

The relation of the quantum yield of CO_2_ assimilation (Ф_*CO2*_) after Valentini^35^ to the operating efficiency of PSII (*F*_q_′/*F*_m_′) under non-photorespiratory conditions can be described as a linear relationship^30^, where *b* is the y axis intercept and *k* is the slope of the relationship between *F*_q_′/*F*_m_′ and Ф_*CO2*_:


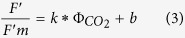


Assuming 4 electrons are necessary for the fixation of a CO_2_ molecule, this can be expressed as:


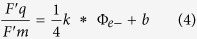


where Φ_e−_ is the quantum yield of total electron flow, which can be rewritten as:


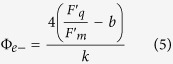


This relationship is assumed to hold in the presence and in the absence of photorespiration, where in the presence of photorespiration it can be used to calculate the total electron flow, which is the sum of electron flow to the reactions of carboxylation (*J*_C_) and oxygenation (*J*_O_)^35^:


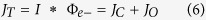


*J*_*C*_ can be calculated by assuming 4 electrons are needed per CO_2_ for carboxylation:


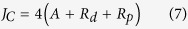


where *R*_p_ is the amount of CO_2_ released by photorespiration. *J*_O_ can be calculated by assuming that 8 electrons are needed per CO_2_ released in photorespiration:


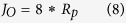


Combining the equations for *J*_*T*_, *J*_*C*_ and *J*_*O*_ then gives:


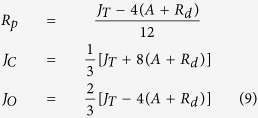


### Leaf transmission and reflectance

Immediately following completion of the gas exchange measurements leaf absorbance (*α*) was measured from 400 to 700 nm, with a dual-channel spectrometer and integrating spheres (SpectroClip-JAZ-TR, Ocean Optics, Oxford, UK). Six spectral measurements of leaf transmittance and reflectance were made per leaf. The average transmittance (τ) and reflectance (R) for each leaf was used to determine *α* = (1 − R − τ). Total absorbance for the PAR spectrum was calculated. Total absorbed light was also calculated by combining the percentage of actinic light emitted by the blue (470 nm) and red (640 nm) LEDs in the leaf gas exchange chamber with *α* for the peak wavelength of the two LED types^36^. The apparent maximum quantum yield (Φ_max_) was then recalculated to give the maximum absolute quantum yield, i.e. the net number of CO_2_ molecules absorbed by the leaf per photon absorbed:


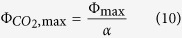


### Intrinsic leaf water use efficiency

Intrinsic leaf water use efficiency (LWUE) was calculated from gas exchange of CO_2_ and H_2_O as the ratio of CO_2_ assimilation (*A*) over stomatal conductance (*g*_*s*_) under 21% O_2_ at photon fluxes of 1000 and 2000 μmol m^−2^ s^−1^. Contrary to the water use efficiency calculated from *A* over transpiration (*E*)^37^, *A*/*g*_*s*_ is considered to be more realistic and comparable between studies, as it is not influenced by changes in leaf to air VPD in the leaf chamber^38^.

## Results

**Figure 1:**
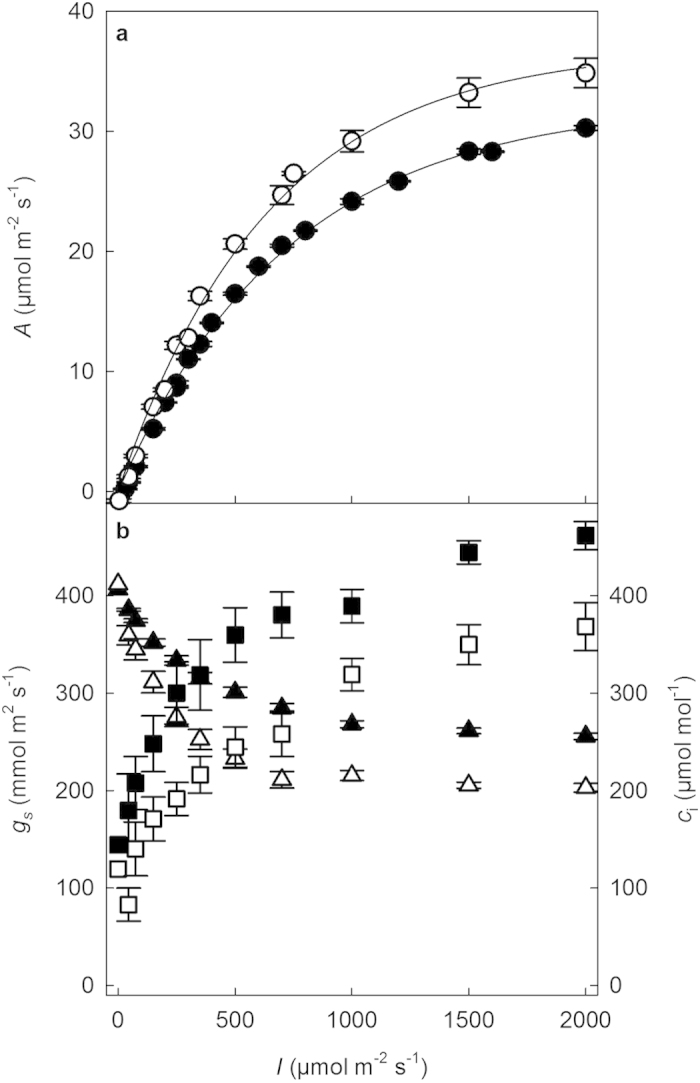
(**a**) Light response of assimilation rate for fully emerged *A. donax* leaves measured at 21% oxygen (closed symbols) and 2% oxygen (open symbols). (**b**) *c*_i_ (triangle) and *g*_s_ (square) at a *c*_*a*_ of 400 μmol mol^−1^, leaf temperature of 25 °C and VPD of between 0.8 and 1.1 kPa (values are means and standard errors, for 5 different plants).

The light response of *A. donax* for *A* at high and low O_2_ showed an increase of *A* when O_2_ was lowered, compared to that at 21% O_2_, indicating the reduction of *A* in air due to photorespiration (Fig. 1a). At both O_2_ concentrations, *A* did not reach a plateau, even at high photon flux. Both the light response of *g*_*s*_ and *c*_i_ were lowered when O_2_ concentration was reduced (Fig. 1b). The response of *g*_*s*_ to light largely scaled with *A*, while *c*_i_ reached a plateau above a photon flux of 600 μmol m^−2^ s^−1^. From the response of *A* to light, parameters were derived (Table 1). The CO_2_ assimilation rate at saturating photon flux (*A*_sat_) was remarkably high at both ambient (30.19 +/−0.32 μmol m^−2^ s^−1^) and low O_2_ concentration (34.86 +/−1.2 μmol m^−2^ s^−1^) whereas the predicted values of *A* at infinite photon flux density as described by the fitted hyperbola were 38.11 +/−0.56 μmol m^−2^ s^−1^ at ambient O_2_ and 43.45 +/−2.08 μmol m^−2^ s^−1^ at low O_2_ concentration. The maximum quantum yield of CO_2_ assimilation (Φ_CO2,max_) at ambient and low O_2_ level was found to be 0.056 (+/−0.0029) and 0.072 (+/−0.0045) respectively, mol CO_2_ (mol absorbed photons)^α1^. The intrinsic leaf water use efficiency (*A*/*g*_*s*_) (LWUE) was 62.9 (+/−2.38) μmol mol^−1^ at sub-saturating photon flux (1000 μmol m^−2^ s^−1^) and 66.0 (+/−1.97) μmol mol^−1^ at near-saturating photon flux (2000 μmol m^−2^ s^−1^).

**Figure 2:**
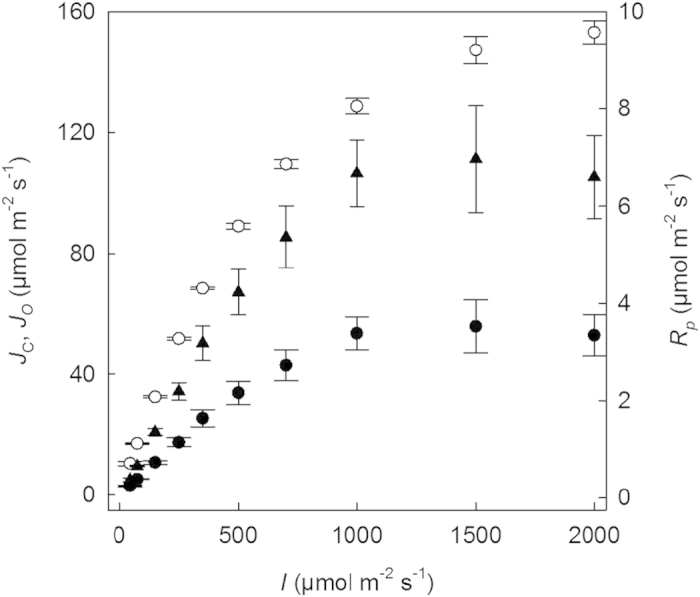
Electron flow to the reaction of carboxylation (*J*_C_) (open circle) and oxygenation (*J*_O_) (closed circle) and the rate of CO_2_ production by photorespiration (*R*_p_) (closed triangle) for fully emerged *A. donax* leaves. Values are corrected for leaf absorbance, 0.89 (±4.47 × 10^−3^) and are calculated from light response of assimilation rate at a *c*_a_ of 400 μmol mol^−1^, leaf temperature of 25 °C and VPD of between 0.8 and 1.1 kPa. (Values are means and standard errors, for 5 different plants).

Under 21% O_2_, the electron flow to oxygenation reactions of Rubisco (*J*_O_) and the rate of CO_2_ production by photorespiration (*R*_*p*_) increased with photon flux, until a plateau was reached at a photon flux of 1000 μmol m^−2^ s^−1^ (Fig. 2.). However, the electron flow to carboxylation (*J*_C_) increased further with increasing photon flux and did not appear to reach a plateau (Fig. 2). It was estimated that *ca*. 25% of electron flow is accounted for by *J*_O_ under saturating light intensity. The ratio of CO_2_ production by photorespiration, *R*_p_, to assimilation (*A*_sat_) was 0.216.

**Figure 3:**
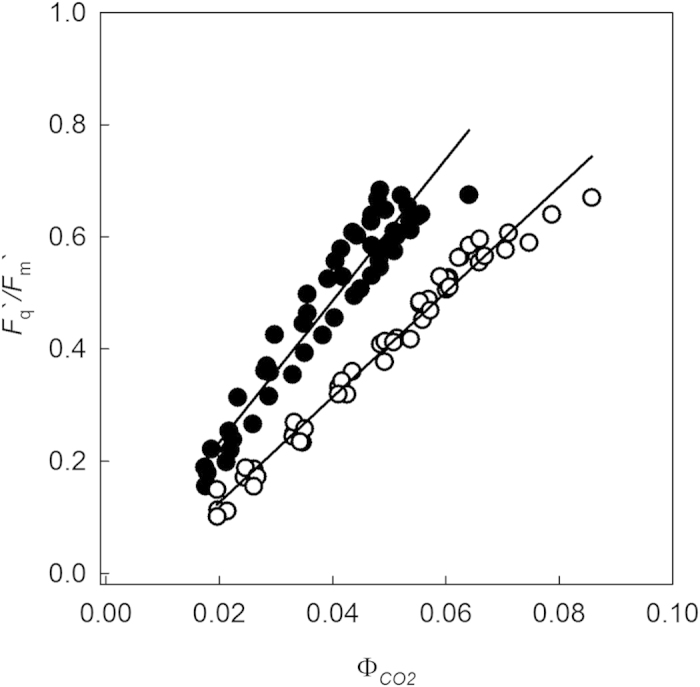
Relationship between operating efficiency of PSII (*F*_q_′/*F*_m_′) and quantum yield of CO_2_ assimilation (Ф_*CO2*_) for *A. donax* calculated from light response of assimilation rate, measured at 21% oxygen (closed circle) and 2% oxygen (open circle) and both at a *c*_*a*_ of 400 μmol mol^−1^, leaf temperature of 25 °C and VPD of between 0.8 and 1.1 kPa. Values are corrected for leaf absorbance, 0.89 (±4.47 × 10^−3^), (values are means and standard errors, for 5 different plants).

Total leaf absorbance from 400 nm to 700 nm was 0.87 (+/− 4.0 × 10^−3^, n = 6). Total leaf absorbance at the peak emitting wavelengths of red (640 nm) and blue (470 nm) was 0.89 (S.E. +/^–^4.47 × 10^−3^, n = 6) and used to calculate *F*_q_′/*F*_m_′ against the quantum yield of CO_2_ assimilation (Ф_*CO2*_) on an absorbed light basis. The relation between *F*_q_′*/F*_m_′ and Ф_*CO2*_ under high and low O_2_ showed significant positive correlations (Fig. 3). The difference in slope of the relation, representing the apparent electron demand to assimilate one CO_2_, at ambient and low O_2_ concentration can be attributed to the presence or absence of photorespiration.

**Figure 4:**
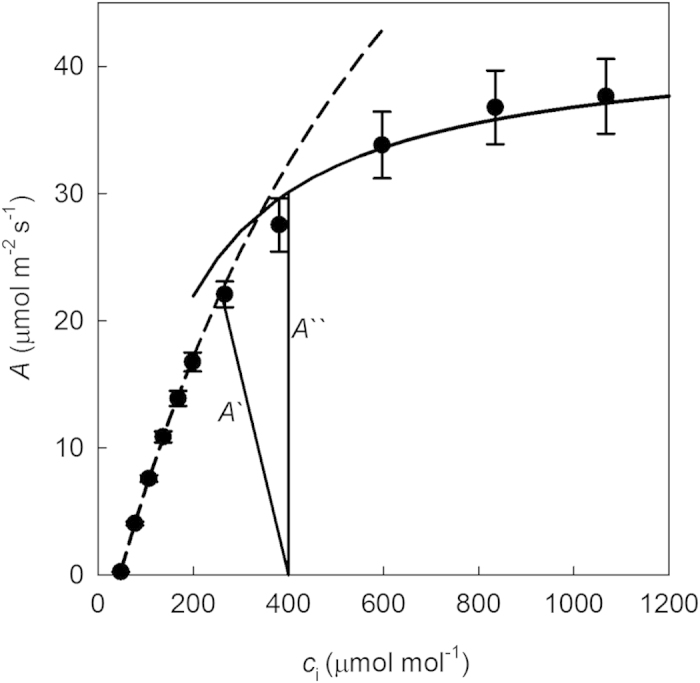
*A* vs *c*_i_ response for *A. donax*. *A′*; the supply function of the estimated limitation placed on *A* by the stomata and leaf boundary layer; *A*′′ the supply function in the absence of any limitation to diffusion of CO_2_ from the atmosphere to the site of carboxylation. Observations were measured at 21% oxygen on fully emerged leaves at a *I* of 1500 μmol m^−2^ s^−1^, leaf temperature of 25 °C and VPD of 0.96 (0.01) kPa and *g*_s_ 0.404 (0.016) mol m^−2^ s^−1^. (Values are means and standard errors, for 5 different plants).

The response of *A* to *c*_i_ for *A. donax* leaves at saturating light intensity was determined at ambient O_2_ concentration (Fig. 4). Subsequently, parameters were derived to describe the *A*/*c*_i_ response and the stomatal limitation (Table 2). The maximum velocity of carboxylation by Rubisco (*V*_c,max_), was 117.8 μmol m^−2^ s^−1^ and the maximum rate of electron transport (*J*_max_) was 213.7 μmol m^−2^ s^−1^. The CO_2_ compensation point, Γ, was 44.2 μmol mol^−1^ and the limitation to *A* imposed by stomata and leaf boundary layer, *l*, was 0.25 (Table 2).

Soil water content between 20 cm and 40 cm soil depth averaged 0.14 m^3^ m^−3^ (+/− 0.01) across the sampling area over the sampling period, with an average soil temperature of 21 ^o^C (+/− 0.9). Stomatal conductance of *A. donax*, measured on ad- and abaxial surfaces of leaves on three different positions along the leaf blade, were highest at noon (12:00) and declined in the afternoon (16:00, Table 3). The highest stomatal conductance was observed in the middle of the leaf at both time points and both surfaces. The greatest decline in stomatal conductance between noon and afternoon was observed in the middle of the leaf, on both adaxial and abaxial surfaces (Table 3). Even though there was a great decline in stomatal conductance, the operating efficiency of PSII (*F*_q_′*/F*_m_′) at noon and in the afternoon in the field did not decrease as much (Table 3). This implies that stomatal conductance was still not greatly limiting for photosynthesis during the afternoon.

## Discussion

Light-saturated capacity for photosynthetic CO_2_ uptake is determined by the maximum rate of carboxylation (*V*_c,max_) and the maximum rate of electron transport, (*J*_max_), depending on the inter-cellular CO_2_ concentration (*c*_*i*_)^20^. Averaged across 109 C3 species, *V*_c,max_ was 64 μmol m^−2^ s^−1^ and *J*_max_ 134 μmol m^−2^ s^−1^ 39, this compares to values found here for *A. donax* of 117.8 and 213.7 μmol CO_2_ m^−2^ s^−1^, respectively, which are almost double the C3 average. These values are for a natural and unfertilized stand. Typically in C3 crops these values will increase with nitrogen status^40^. However, the values found here for *A. donax* are high even compared to fertilized or N-fixing productive crops: *Phalaris arundinacae* (83.9 and 150.6 μmol m^−2^ s^−1^)^41^, *Triticum aestivum* (59 and 139 μmol m^−2^ s^−1^)^40^, *Oryza sativa* (91 and 190 μmol m^−2^ s^−1^ 42, *Helianthus annuus* (29 and Heinz walz μmol m^−2^ s^−1^)^43^, *Glycine max* (83 and 160 μmol m^−2^ s^−1^)^44^, and *Phaseolus vulgaris* (88 and 178 μmol m^−2^ s^−1^)^45^. This highlights the high light-saturated maximum photosynthetic capacity of *A. donax*. Compared to other tall grass species considered as bioenergy sources, *A. donax* shows high *A* in full sunlight. Figure 1 shows an average *A* of 30.2 μmol m^−2^ s^−1^ at 25 °C, this compares to values at this temperature of 28 μmol m^−2^ s^−1^ for *M. x giganteus*^46^, 24 μmol m^−2^ s^−1^ in field grown stands of *Panicum virgatum* (switchgrass)^16^, and 22 μmol m^−2^ s^−1^ for the C3 bioenergy grass, *Phalaris arundinacea* (Reed canary grass)^47^.

Is this *A* achieved by lower losses to photorespiration in *A. donax*? If we assume “average” Rubisco kinetic properties as described by Brooks and Farquhar^48^ for spinach, then the CO_2_ photosynthetic compensation point (Γ*) would be 42.5 μmol mol^−1^ at 25 °C. Using this value, with the measured dark respiratory rate (*R*_d_) of 2 μmol m^−2^ s^−1^, average *A* of 30.19 μmol m^−2^ s^−1^ and *c*_i_ of 255 μmol m^−2^ s^−1^ at a photon flux of 2000 μmol m^−2^ s^−1^ (Fig. 1), the photorespiratory rate of CO_2_ release (*R*_p_) would be 6.3 μmol m^−2^ s^−1^, following the equations of Farquhar *et al.*^20^. This calculated rate, based on properties of spinach, is very close to the rate derived here for *A. donax* by combining fluorescence and gas exchange measurements (Fig. 2) via the equations of Valentini *et al.*^35^. This suggests that as a proportion of net photosynthesis, photorespiratory loss of carbon is just as great in *A. donax* as in other C3 species.

Is the rate of CO_2_ assimilation in limiting light high in *A. donax*? Light limited photosynthesis is determined by the efficiency with which the leaf can absorb incident light (*α*), and the maximum absolute quantum yield of CO_2_ assimilation (Φ_CO2,max_), i.e. the initial slope of the response of *A* to *I*, corrected for absorptance (*α*). The value is therefore the maximum ratio of net absorbed CO_2_ molecules to absorbed photons. The Φ_CO2,max_ determined for *A. donax* here in normal air at 25 °C was 0.056 mol mol^−1^. Osborne and Garrett^49^ similarly measured Φ_CO2,max_ by combining gas exchange and integrating sphere measurements across a range of C3 herbage grasses and cereals, covering different ploidy levels and cultivars in normal air and at 25 °C. They reported an average Φ_CO2,max_ of 0.051, with a range of 0.047 to 0.055 across these C3 grasses. So this does place the values here for *A. donax* at the upper end of this range and 8% higher than the average. The realized efficiency of light limited photosynthesis will be the product of Φ_CO2,max_ and the absorptance of the leaf (*α*). In surveying a wide range of healthy leaves of C3 species from tropical to polar habitats, Long *et al.*^50^, found an average *α* of 0.80, and a range across species from 0.65 to 0.90, similarly measured in an integrating sphere. By comparison, the *α* for *A. donax* of 0.89 therefore appears at the upper end of this range and 14% higher above the average. The product of the indicated above average Φ_CO2,max_ and *α* therefore represents a 24% increase in CO_2_ uptake per unit incident light, under light-limiting conditions.

Does *A. donax* achieve high photosynthetic rates by minimizing stomatal limitation? The stomatal limitation imposed on photosynthesis, *l,* for *A. donax* found in this study (0.25 or 25%) was higher than for other C3 plants, which varied from 0.137 to 0.217^51^,^52^,^53^,^54^. This indicates that *A. donax* does not achieve its high photosynthetic rates through a high stomatal conductance. Indeed *c*_i_/*c*_a_ at full sunlight (2000 μmol m^−2^ s^−1^) as a measure of the balance between stomatal conductance and assimilation was 0.64. This is almost 11% less than the average *c*_i_/*c*_a_ of 0.72 for a range of C3 species^19^. A lower *c*_i_/*c*_a_ requires a lower stomatal conductance relative to the photosynthetic rate, indicating a higher leaf level water use efficiency for a given leaf-air water vapour pressure deficit than the average for C3 species. So, while stomatal conductance may appear high (Fig. 1b), the low values of *l* and *c*_i_/*c*_a_ in fact show that conductance is low relative to the high rates of leaf CO_2_ uptakes for a C3 plant. Nevertheless its high light-saturated and light-limited photosynthetic rates can still only be achieved at the expense of considerable transpiration. The relatively high stomatal conductance maintained into the late afternoon (Table 3) suggests that the plant may be able to tap deep water resources even in its native Mediterranean in the late summer. The LWUE of *A. donax* in this study (62.9 to 66.0 μmol mol^−1^) was higher than generally found for herbaceous species (43 μmol mol^−1^) and more similar to LWUE found for evergreen shrubs and deciduous trees (64 and 66 μmol mol^−1^, respectively)^55^. However, the LWUE of *A. donax* was still much lower than for C4 species such as *Miscanthus* and switchgrass (115 and 107 μmol mol^−1^)^16^. The high productivity of *A. donax* does resemble and exceeds that of C4 plants, however its LWUE is much lower and clearly C3-like. This supports the notion that, as discussed above, *A. donax* is able to achieve its high photosynthetic rates with substantial transpiration, but is still more efficient than most C3 species. This is interesting when considering the diversity of habitat distribution that *A. donax* is found in, which ranges from very wet loam to relatively dry sandy soils.

Overall this study has found that the photosynthetic capacity of *A. donax* in full sunlight is high compared to other C3 species, and comparable to C4 bioenergy grasses. This is not the result of lower photorespiratory rates, but rather a high capacity for both RuBP-limited and RuBP-saturated photosynthesis, as evidenced by values of both *V*_c,max_ and *J*_max_ that are near double the average for C3 species. High photosynthetic rates were not achieved through a high stomatal conductance, in fact stomatal limitation was found to be greater not less than in other C3 species. Light-limited photosynthesis, which will determine carbon uptake during periods of low light flux, around dawn and dusk, and during heavy cloud, as well as in the lower canopy may be aided by relatively high maximum quantum yields of CO_2_ assimilation and high leaf absorptances.

## Additional Information

**How to cite this article**: Webster, R. J. *et al.* High C3 photosynthetic capacity and high intrinsic water use efficiency underlies the high productivity of the bioenergy grass *Arundo donax. Sci. Rep.* **6**, 20694; doi: 10.1038/srep20694 (2016).

## Supplementary Material

Supplementary Information

## Figures and Tables

**Table 1:** Parameters derived from photosynthetic light response curves calculated with incident (*I*) and absorbed photon flux (*αI*); replication and conditions of measurement as given in Fig. 1.

*Photosynthetic light response curves*	21% Oxygen	2% Oxygen
*A*_sat_ (μmol CO_2_ m^−2^ s^−1^)	38.11 (0.56)	43.45 (2.08)
Ф_max_ (mol CO_2_ mol photons^−1^)	0.050 (0.003)	0.064 (0.004)
Φ_CO2,max_ (mol CO_2_ mol photons^−1^)	0.056 (0.003)	0.072 (0.004)
*R*_d_ (μmol CO_2_ m^−2^ s^−1^)	1.471 (0.15)	1.581 (0.16)
θ (−)	0.543 (0.06)	0.601 (0.08)
LCP (μmol m^−2^ s^−1^)	29.88 (2.14)	24.89 (1.63)

mean, (SE), n = 5.

**Table 2:** Parameters derived from photosynthetic intracellular CO_2_ (*c*_i_) response curves; replication and conditions of measurement as given in Fig. 4.

*Photosynthetic intercellular-CO*_*2*_*response*	21% Oxygen
*V*_c,max_ (μmol CO_2_ m^−2^ s^−1^)	117.8 (8.8)
*V*_c,max25_ (μmol CO_2_ m^−2^ s^−1^)	95.0 (6.9)
*J*_max_ (μmol CO_2_ m^−2^ s^−1^)	213.7 (21.2)
*J*_max25_ (μmol CO_2_ m^−2^ s^−1^)	184.0 (18.1)
Γ (μmol mol^−1^)	44.2 (0.95)
*l* (−)	0.25 (0.05)

mean, (SE), n = 5.

**Table 3:** Stomatal conductance (mmol m^−^^2^ s^−^¹) at mid and late afternoon, on abaxial and adaxial leaf surfaces with measurements along the leaf surface from the stem to the leaf tip.

Time	LeafSurface	Stem	Middle	Tip	*F*_q_′/*F*_m_′
12:00	Abaxial	399 (61)	538 (72)	355 (25)	
12:00	Adaxial	293 (30)	422 (35)	326 (64)	0.63 (0.03)
16:00	Abaxial	231 (24)	373 (77)	341 (87)	
16:00	Adaxial	110 (10)	210 (18)	160 (28)	0.51 (0.08)

(Values are means and standard errors for 4 or 5 individual plants). The operating efficiency of PSII (*F*_q_′/*F*_m_′) at mid and late afternoon, on the middle adaxial leaf surfaces.
