# Supplementary material for: High C3 photosynthetic capacity and high intrinsic water use efficiency underlies the high productivity of the bioenergy grass Arundo donax
Source: Sci Rep. 2016 Feb 10;6:20694. doi: 10.1038/srep20694 (PMC4748246; doi:10.1038/srep20694)
Supplement: Supplementary Information [file srep20694-s1.pdf]

Supplementary data.

High C3 photosynthetic capacity and high intrinsic water use efficiency underlies the high productivity of the bioenergy grass *Arundo donax*.

*Richard J. Webster, Steven M. Driever, Johannes Kromdijk, Justin McGrath, Andrew D. B. Leakey, Katharina Siebke, Tanvir Demetriades-Shah, Steve Bonnage, Tony Peloe, Tracy Lawson, Stephen P. Long.

Figure S1. PAR (μmol m^{-2} s^{-1}) (solid grey line), Air Temperature (^{o}C) (dashed black line) and VPD (kPa) (solid black line) of *Arundo donax* stand at Quinta de São Pedro during the sampling period. Arrows indicate the times of stomatal conductance and the operating efficiency of PSII (F_q\`/$F_m$\`) measurements as shown in Table 3.

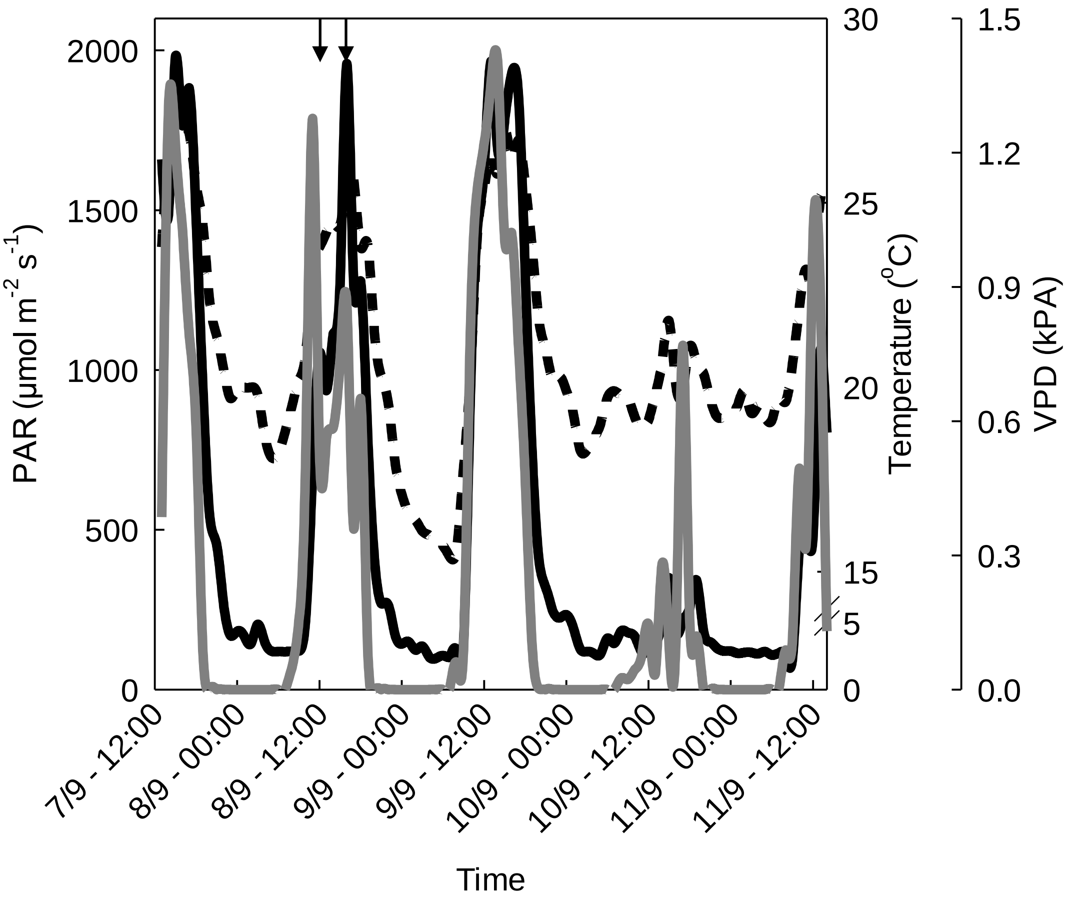

Figure S1.
